# Supplementary material for: Correlating STED and synchrotron XRF nano-imaging unveils cosegregation of metals and cytoskeleton proteins in dendrites
Source: eLife. 2020 Dec 8;9:e62334. doi: 10.7554/eLife.62334 (PMC7787660; doi:10.7554/eLife.62334)
Supplement: Supplementary file 1. — Analysis of chemical elements content for 21 regions showing zinc and tubulin co-localization, expressed in ng.mm−2 and in atoms.nm−2 (mean ± SD, n = 21). [file elife-62334-supp1.docx]

|  | **Phosphorus** | **Sulfur** | **Potassium** | **Zinc** |
| --- | --- | --- | --- | --- |
| ng.mm^-2^ | 0.607 ± 0.169 | 0.211 ± 0.068 | 0.167 ± 0.080 | 0.010 ± 0.001 |
| atoms.nm^-2^ | 11.81 ± 3.29 | 3.97 ± 1.28 | 2.57 ± 1.23 | 0.09 ± 0.01 |
