# Supplementary material for: Correlating STED and synchrotron XRF nano-imaging unveils cosegregation of metals and cytoskeleton proteins in dendrites
Source: eLife. 2020 Dec 8;9:e62334. doi: 10.7554/eLife.62334 (PMC7787660; doi:10.7554/eLife.62334)
Supplement: Supplementary file 2. [file elife-62334-supp2.docx]

| **Figure** | **Data** | **Population size** | **Distribution normality**  *(Shapiro-Wilk test)*  *p<0.05 normal distribution not met. If p>0.05, then check for homogeneity of variance (Bartlett's test)*  *p<0.05 homogeneity not met* | **Comparison test** |
| --- | --- | --- | --- | --- |
| **Fig. 6d** | β-tubulin fluorescence intensities (normalized) | Control group  n = 62  TPEN group  n = 62  TPEN + Zn group  n = 68 | Shapiro-Wilk test:  Control group  p = 4.3e-3  TPEN group  p = 4.035e-07    TPEN + Zn group  p = 1.514e-06 | Kruskal-Wallis:  p-value = 3.1e-07  Dunn’s test, p-values adjusted with the Holm method  Ctrl vs TPEN  p.adj = 1.1e-4  Ctrl vs TPEN+Zn  p.adj = 2.8e-1  TPEN vs TPEN+Zn  p.adj = 4.8e-7 |
| **Fig. 6e** | F-actin fluorescence intensities (normalized) | Control group  n = 64  TPEN group  n = 59  TPEN + Zn group  n = 67 | Shapiro-Wilk test:  Control group  p = 5.9e-3  TPEN group  p = 2.7e-10  TPEN + Zn group  p = 5.0e-4 | Kruskal-Wallis:  p-value < 2.2e-16  Dunn’s test, p-values adjusted with the Holm method  Ctrl vs TPEN  p.adj = 1.1e-14  Ctrl vs TPEN+Zn  p.adj = 3.9e-2  TPEN vs TPEN+Zn  p.adj = 9.4e-23 |
| **Fig. 6 figure supplement 1A** | β-tubulin fluorescence intensities (normalized) | Control group  n = 34  TPEN group  n = 35 | Shapiro-Wilk test:  Control group  p = 1.6e-3  TPEN group  p = 1.3e-1 | Mann-Whitney test:  Ctrl vs TPEN  p = 1.2e-3 |
| **Figure 6-figure supplement 1B** | β-tubulin fluorescence intensities (normalized) | Control group  n = 41  TPEN group  n = 45  TPEN + Zn group  n = 42 | Shapiro-Wilk test:  Control group  p = 6.1e-1  TPEN group  p = 3.6e-1  TPEN + Zn group  p = 1.4e-1  Bartlett's test:  p-value = 7.5e-2 | ANOVA:  p=7.5e-8  Tukey post-hoc test:  Ctrl vs TPEN  p.adj = 4.6e-8  Ctrl vs TPEN+Zn  p.adj = 5.7e-2  TPEN vs TPEN+Zn  p.adj = 8.8e-4 |
| **Figure 6-figure supplement 1C** | β-tubulin fluorescence intensities (normalized) | Control group  n = 135  TPEN group  n = 116  TPEN + Zn group  n = 120 | Shapiro-Wilk test:  Control group  p = 4.7e-7  TPEN group  p = 7.6e-8  TPEN + Zn group  p = 2.5e-8 | Kruskal-Wallis:  p-value = 6.5e-12  Dunn’s test, p-values adjusted with the Holm method  Ctrl vs TPEN  p.adj = 3.0e-12  Ctrl vs TPEN+Zn  p.adj = 1.2e-4  TPEN vs TPEN+Zn  p.adj = 2.3e-3 |
| **Figure 6-figure supplement 1D** | F-actin fluorescence intensities (normalized) | Control group  n = 34  TPEN group  n = 35 | Shapiro-Wilk test:  Control group  p = 1.7e-7  TPEN group  p = 1.4e-2 | Mann-Whitney test:  Ctrl vs TPEN  p = 2.7e-2 |
| **Figure 6-figure supplement 1E** | F-actin fluorescence intensities (normalized) | Control group  n = 41  TPEN group  n = 44  TPEN + Zn group  n = 42 | Shapiro-Wilk test:  Control group  p = 4.2e-1  TPEN group  p = 8.6e-4  TPEN + Zn group  p = 2.0e-1 | Kruskal-Wallis:  p-value = 2.1e-11  Dunn’s test, p-values adjusted with the Holm method  Ctrl vs TPEN  p.adj = 2.5e-12  Ctrl vs TPEN+Zn  p.adj = 1.1e-3  TPEN vs TPEN+Zn  p.adj = 2.0e-4 |
