# Supplementary material for: Correlating STED and synchrotron XRF nano-imaging unveils cosegregation of metals and cytoskeleton proteins in dendrites
Source: eLife. 2020 Dec 8;9:e62334. doi: 10.7554/eLife.62334 (PMC7787660; doi:10.7554/eLife.62334)
Supplement: Transparent reporting form [file elife-62334-transrepform.pdf]

eLIFE
1st Floor
24 Hills Road
Cambridge CB2 1JP, UK
P 01223 855340
W elifesciences.org
T @elife

eLife's transparent reporting form

We encourage authors to provide detailed information *within their submission* to facilitate the interpretation and replication of experiments. Authors can upload supporting documentation to indicate the use of appropriate reporting guidelines for health-related research (see EQUATOR Network), life science research (see the BioSharing Information Resource), or the ARRIVE guidelines for reporting work involving animal research. Where applicable, authors should refer to any relevant reporting standards documents in this form.

If you have any questions, please consult our Journal Policies and/or contact us: editorial@elifesciences.org.

Sample-size estimation

- You should state whether an appropriate sample size was computed when the study was being designed
- You should state the statistical method of sample size computation and any required assumptions
- If no explicit power analysis was used, you should describe how you decided what sample (replicate) size (number) to use

Please outline where this information can be found within the submission (e.g., sections or figure legends), or explain why this information doesn't apply to your submission:

This information applies to the quantitative immunofluorescence experiments (Figure 6 and Figure 6-supplement 1). The sample size was limited by the duration of the experiments since all fluorescence images had to be acquired on the same day to avoid fluorescence changes due to storage. 20-25 neurons were observed per coverslip, 2-4 coverslips per condition. See '*Methods. Quantitative fluorescence microscopy and data analysis'* for details.

Replicates

- You should report how often each experiment was performed
- You should include a definition of biological versus technical replication
- The data obtained should be provided and sufficient information should be provided to indicate the number of independent biological and/or technical replicates
- If you encountered any outliers, you should describe how these were handled
- Criteria for exclusion/inclusion of data should be clearly stated
- High-throughput sequence data should be uploaded before submission, with a private link for reviewers provided (these are available from both GEO and ArrayExpress)

Please outline where this information can be found within the submission (e.g., sections or figure legends), or explain why this information doesn't apply to your submission:

The comparison of the exposure conditions was repeated in 3 independent experiments, technical replicates (Figure. 6 and Figure 6-supplement 1). For each technical replicate, and for each of exposure condition, between 2 and 4 coverslips were observed, each of these coverslips being sampled from the dissection of a different animal (biological replicates). See '*Methods*' section.

eLife Sciences Publications, Ltd is a limited liability non-profit non-stock corporation incorporated in the State of Delaware, USA, with company number 5030732, and is registered in the UK with company number FC030576 and branch number BR015634 at the address 1st Floor, 24 Hills Road, Cambridge CB2 1JP | August 2014

Statistical reporting

- Statistical analysis methods should be described and justified
- Raw data should be presented in figures whenever informative to do so (typically when N per group is less than 10)
- For each experiment, you should identify the statistical tests used, exact values of N, definitions of center, methods of multiple test correction, and dispersion and precision measures (e.g., mean, median, SD, SEM, confidence intervals; and, for the major substantive results, a measure of effect size (e.g., Pearson's r, Cohen's d)
- Report exact p-values wherever possible alongside the summary statistics and 95% confidence intervals. These should be reported for all key questions and not only when the p-value is less than 0.05.

Please outline where this information can be found within the submission (e.g., sections or figure legends), or explain why this information doesn't apply to your submission:

This information is detailed in Supplementary File 2, statistical analysis of data.

(For large datasets, or papers with a very large number of statistical tests, you may upload a single table file with tests, Ns, etc., with reference to sections in the manuscript.)

Group allocation

- Indicate how samples were allocated into experimental groups (in the case of clinical studies, please specify allocation to treatment method); if randomization was used, please also state if restricted randomization was applied
- Indicate if masking was used during group allocation, data collection and/or data analysis

Please outline where this information can be found within the submission (e.g., sections or figure legends), or explain why this information doesn't apply to your submission:

This information applies to the quantitative immunofluorescence experiments (Figure 6 and Figure 6-supplement 1). Samples were allocated randomly for each group of exposure conditions.

Additional data files ("source data")

- We encourage you to upload relevant additional data files, such as numerical data that are represented as a graph in a figure, or as a summary table
- Where provided, these should be in the most useful format, and they can be uploaded as "Source data" files linked to a main figure or table
- Include model definition files including the full list of parameters used
- Include code used for data analysis (e.g., R, MatLab)
- Avoid stating that data files are "available upon request"

Please indicate the figures or tables for which source data files have been provided:

eLife Sciences Publications, Ltd is a limited liability non-profit non-stock corporation incorporated in the State of Delaware, USA, with company number 5030732, and is registered in the UK with company number FC030576 and branch number BR015634 at the address 1st Floor, 24 Hills Road, Cambridge CB2 1JP | August 2014

Synchrotron datasets (SXRF and PCI images) are available from the ESRF data portal in open mode in two datasets. Data sets are associated to the following DOI numbers: doi:10.15151/ESRF-ES-162248067 and doi:10.15151/ESRF-ES-101127303. The link to the first dataset is https://data.esrf.fr/investigation/162248067/datasets, sign in as anonymous to access data. The link to the second dataset is https://data.esrf.fr/investigation/101127303/datasets, sign in as anonymous then click on the zoom button to reveal the download button. Source data for Figure 1 are available at https://data.esrf.fr/investigation/162248067/datasets; datasets M20_zone67_nfp3_015nm and M20_zone67_fine01. Source data for Table 1 are included in file Table 1—source data 1. Source data for Figure 2 are available at https://data.esrf.fr/investigation/101127303/datasets; datasets TA15_neu64_fine2 and TA15_neu64_fine5. Synchrotron XRF data for Figure 2—figure supplement 1 are available at https://data.esrf.fr/investigation/101127303/datasets; datasets TA15_neu64_fine4 and TA15_neu64_fine3. Data for Pearson's correlation coefficients of Figure 2—figure supplement 1 panel h are provided in Figure 2—figure supplement 1—source data 1. Data for Pearson's correlation coefficients of Figure 2—figure supplement 1 panel o are provided in Figure 2—figure supplement 1—source data 2. Source data for Figure 3 are available at https://data.esrf.fr/investigation/162248067/datasets; datasets M8_neur43_sted44_nfp_015nm and M8_neu43_fine03. Synchrotron XRF data for Figure 3—figure supplement 1 are available at https://data.esrf.fr/investigation/101127303/datasets; dataset SiTA1_neu7_fine01. Source data for Figure 4 are available at https://data.esrf.fr/investigation/101127303/datasets; dataset TA15_neu71_fine01. Data for Pearson's correlation coefficients are included in file Figure 4—source data 1. Synchrotron XRF and PCI data for Figure 4—figure supplement 1 are available at https://data.esrf.fr/investigation/162248067/datasets; datasets M20_zone67_fine01, M20_zone67_fine02, and M20_zone67_fine06. Source data for Figure 5 are available at https://data.esrf.fr/investigation/101127303/datasets; datasets TA15_neu26_fine 01 and TA15_neu23_fine02. Synchrotron XRF data for Figure 5—figure supplement 1 are available at https://data.esrf.fr/investigation/162248067/datasets; datasets M20_zone67_nfp3_015nm and M20_zone67_fine01. F-actin data for Figure 6 are available in file Figure 6—source data 1. β-tubulin data for Figure 6 are available in file Figure 6—source data 2. F-actin data for Figure 6-figure supplement 1 are available in file Figure 6—figure supplement 1—source data 1. Tubulin data for Figure 6-figure supplement 1 are available in file Figure 6—figure supplement 1—source data 2. Supplementary file 1 raw data provided in Source data 1.

eLife Sciences Publications, Ltd is a limited liability non-profit non-stock corporation incorporated in the State of Delaware, USA, with company number 5030732, and is registered in the UK with company number FC030576 and branch number BR015634 at the address 1st Floor, 24 Hills Road, Cambridge CB2 1JP | August 2014
